# Intermittent Ethanol during Adolescence Leads to Lasting Behavioral Changes in Adulthood and Alters Gene Expression and Histone Methylation in the PFC

**DOI:** 10.3389/fnmol.2017.00307

**Published:** 2017-09-26

**Authors:** Jennifer T. Wolstenholme, Tariq Mahmood, Guy M. Harris, Shahroze Abbas, Michael F. Miles

**Affiliations:** ^1^Department of Pharmacology and Toxicology, Virginia Commonwealth University, Richmond, VA, United States; ^2^VCU Alcohol Research Center, Virginia Commonwealth University, Richmond, VA, United States; ^3^Department of Human and Molecular Genetics, Virginia Commonwealth University, Richmond, VA, United States

**Keywords:** adolescent, ethanol, genomics, prefrontal cortex, epigenetics

## Abstract

Adolescents primarily consume alcohol in binges, which can be particularly harmful to the developing frontal cortex and increase risk for an adult alcohol use disorder. We conducted a study investigating immediate and long lasting changes to the prefrontal cortex (PFC) transcriptome to determine the molecular mechanisms underlying adult ethanol behavioral sensitivity following binge ethanol in adolescence. DBA/2J mice were orally dosed with 4 g/kg ethanol intermittently from day 29 to 42. Adolescent mice were tested for anxiety-like behavior and ethanol sensitivity using the loss of righting reflex task. As adults, mice were tested for cognitive changes using the novel object recognition task, ethanol-induced anxiolysis and ethanol sensitivity. Adolescent binge ethanol altered ethanol sensitivity in young mice and led to lasting memory deficits in the object recognition test and greater ethanol sensitivity in adulthood. Using genomic profiling of transcripts in the PFC, we found that binge ethanol reduced myelin-related gene expression and altered chromatin modifying genes involved in histone demethylation at H3K9 and H3K36. We hypothesize that ethanol’s actions on histone methylation may be a switch for future transcriptional changes that underlie the behavioral changes lasting into adulthood.

## Introduction

Alcohol is the most commonly abused intoxicant among adolescents with 8.7 million underage youth reporting consumption in the past month (Center for Behavioral Health Statistics Quality, 2014). Remarkably, 90% of the alcohol consumed by American youths under the age of 21 is in the form of binge drinking, consuming four or more drinks in a few hours (Center for Behavioral Health Statistics Quality, 2014). Binge drinking in adolescence can heighten sensitivity to the rewarding aspects of alcohol and diminish sensitivity to its aversive effects enabling teens to consume larger amounts of alcohol with fewer negative effects of intoxication (Spear, 2000). This shift in the balance of rewarding and aversive effects of ethanol could increase risk for progression to alcohol use disorders (AUD). Indeed, an early onset age of drinking increases risk for adult AUD nearly fourfold (Grant and Dawson, 1997; Grant, 1998).

Consuming alcohol in adolescence can be particularly harmful, as it may delay or disrupt critical ongoing neurodevelopment with profound consequences in adult brain structure, connection and function. In particular, prefrontal cortex (PFC) is a brain region undergoing dramatic changes in structure and synaptic connectivity during adolescence (Spear, 2000) and shows molecular and structural alterations with ethanol exposure (De Bellis et al., 2005; Pfefferbaum et al., 2006; Medina et al., 2008; Kroenke et al., 2014). The PFC also exerts important top-down or executive control over other brain regions that mediate approach and positively reinforce drug seeking, as well as regions that mediate aversion and negatively reinforce drug seeking (Peters et al., 2009; Koob and Volkow, 2010). Altered connectivity between these structures, or deficits in executive control may lead to loss of control over attention and emotion and can lead to increased engagement in risky behaviors such as binge drinking. Ongoing frontal cortex myelination and synaptic pruning in adolescence may make binge drinkers particularly vulnerable to long-term consequences, such as increases in ethanol sensitivity, sensitization, consumption and long-term cognitive deficits (Spear, 2000; Coleman et al., 2014; Vargas et al., 2014; Beaudet et al., 2016).

Ethanol exposure, either during adolescence or adulthood, is often associated with reduced white matter in the frontal cortex, cortical atrophy and cognitive impairments. White matter degradation (Bava et al., 2013; Luciana et al., 2013) and disrupted myelin gene and protein expression are frequently found in frontal, motor and limbic brain regions of alcoholics (Lewohl et al., 2000). In adolescents with an AUD, neuroimaging studies show decreased white matter in the frontal cortex is associated with increasing ethanol consumption (De Bellis et al., 2005). In adult C57BL/6J or DBA/2J mice, acute or chronic ethanol exposure causes prominent changes in PFC gene expression, including multiple myelin-related genes (Kerns et al., 2005; Farris and Miles, 2013). Rodent models of adolescent binge ethanol also demonstrate frontal neurodegeneration (Crews et al., 2000), loss of neurogenesis (Crews et al., 2006), decreased myelin fiber density (Montesinos et al., 2014; Vargas et al., 2014) and volume reduction in the basal forebrain and other regions in adulthood (Coleman et al., 2011; Gass et al., 2014). Intermittent ethanol during adolescence leads to deficits in reversal learning (Coleman et al., 2014) and novel object recognition (Montesinos et al., 2014). In rats, chronic intermittent ethanol during adolescence impaired recognition memory in adulthood (Pascual et al., 2007; Vetreno and Crews, 2015). Together, these data suggest that adolescent ethanol imparts long lasting changes in brain structure and function, particularly regarding PFC and myelin. The molecular mechanisms underlying the development of these ethanol-induced alterations, and how they lead to lasting behavioral changes, however, remain elusive.

Ethanol exposure also results in changes in the regulation of gene transcription through epigenetic modifications to chromatin and histones. Epigenetic modifications to histones positively and negatively regulate gene expression and may be one mechanism underlying the long lasting cognitive deficits and behavioral sensitivity to ethanol. Indeed, adolescent intermittent ethanol exposure leads to global alterations in histone methylation (Kyzar et al., 2016), histone acetylation and histone deacetylase expression in the central nucleus of the amygdala (Pandey et al., 2015) or PFC (Pascual et al., 2012). Systemic administration of histone deacetylase inhibitors attenuated anxiety-like behavior and increased ethanol consumption (Pandey et al., 2015) and enhanced the acquisition, extinction and reinstatement of ethanol conditioned place aversion (Pascual et al., 2012). Adults were not affected by HDAC inhibition (Pascual et al., 2012; Pandey et al., 2015), suggesting that lasting behavioral responses to adolescent ethanol can be modulated by chromatin regulation.

The molecular mechanisms underlying ethanol-induced persistent changes in PFC development are currently unknown. We hypothesize that adolescent ethanol causes persistent alterations in PFC gene expression networks, contributing to behavioral alterations during adolescence and adulthood. Here, we use an adolescent binge ethanol exposure model in DBA/2J mice to assess behavioral responses in both adolescence and adulthood. We investigated the molecular basis for these identified changes by performing genome-wide expression profiling of PFC during both adolescence and adulthood, and epigenetic studies on global histone methylation. Our studies identified strikingly different immediate and long-lasting transcriptional responses to adolescent ethanol exposure and implicate alterations in histone methylation in the long-lasting behavioral responses to adolescent ethanol.

## Materials and Methods

### Animals

Male and female DBA/2J mice from Jackson Laboratory arrived in the Virginia Commonwealth University vivarium at postnatal day 22 (Bar Harbor, ME, United States). DBA/2J mice were used since they are known to have robust behavioral responses to acute ethanol (Dudek et al., 1991; Phillips et al., 1994; Linsenbardt et al., 2009) and have more prominent changes in myelin-related gene expression after acute ethanol in adults (Kerns et al., 2005) as compared to the more commonly used C57BL6 mice. We focus on behavioral responses to acute ethanol because an individual’s level of response to acute ethanol is a well-established predictor of risk for developing AUD (Schuckit and Smith, 1996). Mice were housed 4/cage in same sex cages in an AALAC-accredited facility under 12-h light/dark cycles with food and water available *ad libitum* for the entire experiment. After a week acclimation to the animal facility, mice were habituated to the gavage procedure with 0.1% saccharin on PND 27 and 28 and then divided into two treatment groups: ethanol treated and control. In experiment 1, DBA/2J males (*n* = 45) and females (*n* = 32) were orally dosed with 4 g/kg ethanol (25% w/v in water by gavage) or water intermittently (2 days on/2 days off) on PND 29, 30, 33, 34, 37, 38, 41, and 42. At this dose, blood ethanol concentrations reached 313 mg/dL 1 h after gavage (*n* = 3 DBA/J2 males at PND 33). These levels are similar to previously published levels in C57BL/6 mice used to model adolescent binge ethanol and display deficits in myelin (Coleman et al., 2011; Montesinos et al., 2014). Mice were then randomly assigned to two groups and were behaviorally tested for ethanol sensitivity as juveniles or adults. As juveniles, mice were tested for basal anxiety-like behavior at PND 43, 24 h after the last ethanol dose, and then for sedation to a high ethanol dose at PND 47. Adult mice (PND66+) were tested for memory deficits with the novel object recognition test, ethanol-induced anxiolysis, and sedation to ethanol. Behavioral tests were separated by at least 4 days.

In experiment 2, a separate cohort of DBA/2J males and females (*n* = 24/sex) were treated intermittently with ethanol exactly as in experiment 1, but were not behaviorally tested. Tissue was collected for gene expression studies at PND 43 (*n* = 22) and PND 66 (*n* = 19). Across both experiments, 14 mice were lost due to issues surrounding gavage (five controls and nine ethanol-treated mice). All surviving animals appeared normal on the basis of grooming behavior, appearance and feeding. Given that it was not an aim of the present study to examine hormonal regulation of the long-term effects of binge ethanol during adolescence, estrous cycles were not controlled or monitored in female mice. All animal housing and care was conducted with the approval of the Virginia Commonwealth University IACUC Committee and in accordance with the NIH Guide for the Care and Use of Laboratory Animals (National Research Council, 2011).

### Anxiety-Like Behavior in the Light–Dark Box

At PND 43, mice were tested for differences in anxiety-like behavior 24 h after their last ethanol/water gavage dose to assess possible ethanol withdrawal-induced anxiety. The light–dark (LD) box conflict model for anxiety-like behavior was conducted using a standard commercial (Med Associates,) apparatus from Med Associates (St. Albans, VT, United States) with an open field (27.3 cm × 27.3 cm × 20.3 cm) divided into equally sized light or dark compartments by a black plastic partition with an opening in the middle to allow for light–dark transitions. Animal position and locomotor activity was monitored by infrared photobeam breaks. Following a 1-h acclimation period to the behavioral room, mice were placed in the center of the light chamber facing the entrance to the dark chamber. Studies consisted of a 5-min test session, initiated once the animal entered the dark compartment. Anxiety-like measures were reported as percent time spent in the light and percent distance traveled in light to control for locomotor activity. An increase in either measure was interpreted as decreased anxiety-like behavior.

In separate groups of animals exposed to ethanol or water gavage as adolescents, mice were tested at PND 66 for acute ethanol-induced anxiolysis in the light–dark model. After 1-h habituation to the behavioral room, mice were injected with 2 g/kg ethanol (10% w/v, i.p.) or an equivalent volume of 0.9% saline. Mice were placed back into their home cage for 5 min to avoid the ethanol locomotor activation phase routinely observed in DBA/2J mice. LD box anxiety-like measures and total locomotor distance were then measured for 5-min as above.

### Loss of Righting Reflex (LORR)

After 1-h habituation to the test room, mice were dosed with a sedating/hypnotic ethanol dose (4 g/kg, i.p.) and returned to home cages until they exhibited loss of righting reflex (LORR), defined by the inability to right themselves three times in 30 s after being in the supine position in a V-shaped trough. LORR was calculated by subtracting time of onset of LORR from recovery time (Costin et al., 2013).

### Novel Object Recognition

We used the novel object recognition task to measure PFC-mediated recognition memory (Warburton and Brown, 2015). Novel object recognition (NOR) involved two phases, a training and a test phase, separated by either a 5-min or a 1-h delay. Mice were habituated to the test cage for 30 min 1 day prior to the NOR task. On the day of the test, mice were habituated to the testing room for 1 h, then to the test cage for 30 min. During the training phase, two of the same objects were placed in opposite corners of an empty clean mouse cage. The mouse was allowed to interact with each object for a 5-min period, then returned to their home cage for a delay period. Half of the mice were tested with a 5-min delay to measure PFC-dependent short term memory. The other half of the mice were tested with a 1-h delay to test longer term, perirhinal cortex mediated recognition memory (Seamans et al., 1995; Warburton and Brown, 2015). During the delay period, one familiar object was replaced by a novel object of similar size. Mice were then placed back into the test arena and allowed to explore both objects. Time in close contact with nose oriented toward the object (<2 cm) was recorded. Scorers were blinded to the sex and treatment of the mice. A discrimination index was calculated by subtracting the time interacting with the familiar object from the time interacting with the novel object divided by the total interaction time. Failure to follow their innate novelty seeking tendencies to spend more time with the novel object was interpreted as impaired recognition memory and PFC dysfunction (Weitzel et al., 2015). Any mouse that did not investigate the objects for more than 10 s during training was not used in the analysis. Two mice in the 1-h test were excluded for this reason.

### RNA Isolation

Total RNA from PFC of experiment 2 was isolated using STAT 60 Reagent (Tel-Test, Friendswood, TX, United States) and RNeasy mini kit (Qiagen, Valencia, CA, United States) according to the manufacturer’s protocol. RNA concentration was determined by absorbance at 260 nm and RNA quality was assessed by Experion automated electrophoresis (Bio-Rad, Hercules, CA, United States) and 28S:18S ratios. All RNA RQI values were >9.0, and 260/280 ratios were between 1.9 and 2.1.

### Microarray Processing and Bioinformatics

Prefrontal cortex RNA from single mice was reverse transcribed and labeled for microarray hybridization using standard kits and protocols from Affymetrix as described (Wolstenholme et al., 2011b). Labeled cDNA was hybridized to GeneChip^®^Mouse Transcriptome Arrays (MTA v 1.0; *n* = 39). Each array was processed through quality control, normalization using Expression Console and the Transcriptome Analysis Center (TAC, Affymetrix), and bioinformatics pipelines previously established (Wolstenholme et al., 2011b). Two microarrays (one PND 43 control female and one PND 66 control female) failed quality control checks, displaying low signal intensity suggesting poor hybridization and were not used in this analysis. Differential gene expression was determined using two separate analysis methods, sstRMA and *S*-score analysis. Arrays from the two ages were run simultaneously but differential expression analysis was performed separately for each age. Signal space transformation RMA (sstRMA) signals were generated with Expression Console (Affymetrix, Santa Clara, CA, United States). Since the TAC program does not perform the desired two-way ANOVA, the limma package (Ritchie et al., 2015) in the Bioconductor suite (Irizarry et al., 2003) was used to generate differential expression with the factors treatment and sex at each age. Significant differentially expressed transcript IDs were called at uncorrected *p* < 0.01. A second analysis was performed using the significance score (*S*-score) algorithm (Zhang et al., 2002; Kennedy et al., 2006), modified for the MTA arrays to determine ethanol-responsive gene expression. This algorithm compares gene expression profiles between treated and control samples at the probe level by calculating relative change in probe pair intensities between treated and control samples and converting probe signals into multiple measurements with equalized errors, which are summed over all probes for a given gene (transcript cluster) to form the significance score (*S*-Score). *S*-scores follow a standard normal distribution across all transcript IDs, with a mean = 0 and standard deviation = 1. *S*-scores were then evaluated by false discovery methods (SAM) for significant deviation from normality. Pairwise *S*-scores were generated for each sex as ethanol treated versus control and averaged together for each biological replicate. Thus, nine ethanol responsive *S*-scores (5 males and 4 females) were generated for the adolescent data and nine *S*-scores were generated for the adults. Data was collapsed over sex to increase power and focus on age-related differences. Significant differentially expressed *S*-scores were determined using One-Class SAM (Saeed et al., 2003) at FDR < 0.05.

Bioinformatics analysis was performed using previously established pipelines (Wolstenholme et al., 2011b, Wolstenholme et al., 2013) and included functional over-representation analysis with Gene Ontology (GO) using the ToppFun suite of tools (Chen et al., 2009) in the ToppGene Suite for gene list enrichment analysis. Candidate gene prioritization and gene network mapping (Wolstenholme et al., 2011b, 2013; Costin et al., 2013; Farris and Miles, 2013; Harenza et al., 2014) was performed using Ingenuity Pathway Analysis^1^ to assess the biological significance and relationships between these genes, based on current scientific literature.

### Quantitative Real-Time PCR

To confirm the microarray findings on candidate genes, PFC total RNA from experiment 2 was reverse transcribed to cDNA using the iScript cDNA kit (Bio-Rad, Hercules, CA, United States). Real-time PCR was performed using the CFX System (Bio-Rad) for SYBR Green-based detection using standard protocols (Wolstenholme et al., 2011a, 2012, 2013). Biological replicate samples were run in triplicate. Quantification of candidate gene expression levels was calculated based on the threshold cycle (*C*_t_) for each well using the provided software and normalized to *PPP2r2p*, *Ublcp1* and *B2M* as endogenous controls. Relative changes in gene expression were normalized to the control male group.

### Global Protein Histone Methylation

To validate the microarray findings on histone methylation, a separate cohort of mice was treated with ethanol during adolescence as described in experiment 2. Histones were extracted from adolescent PFC tissue at PND 43 (*n* = 3–4/group) using the Total Histone Extraction kit (Epigentek, Farmingdale, NY, United States) according to manufacturer directions. Global protein methylation levels of H3K9 and H3K36 were determined using a colorimetric ELISA assay using the EpiQuik Global Pan-Methyl histone H3-K9 and H3-K36 quantification kits (Epigentek, Farmingdale, NY, United States) per manufacturer’s protocols. Absorbance was determined using a spectrophotometer at 450 nm. The results were calculated using a standard curve following the manufacturer’s instructions, and protein levels were expressed as ng/μg.

### Statistics

Behavioral assays, quantitative rtPCR expression data and histone methylation data were analyzed using Two-Way ANOVA with treatment and sex as factors followed by Student–Newman–Keuls *post hoc* tests for significance. *P*-values less than 0.05 were considered significant. Three-way ANOVAs were used to analyze ethanol-induced anxiolysis in the light–dark box with SNK *post hoc* tests. Statistics and bioinformatics used for genomic expression data are described above.

## Results

### Binge Ethanol during Adolescence Does Not Alter Body Weight

Binge ethanol treatment in DBA/2J mice during adolescence did not significantly alter body weight during the treatment period. All groups significantly increased body weight over the course of the treatment [main effect of time, Expt.1: *F*_(10,733)_ = 386.05, *p* < 0.001 and Expt. 2: *F*_(17,715)_ = 359.673, *p* < 0.001]. Two-way repeated measures ANOVAs only showed significant differences in body weights between males and females [Expt.1: *F*_(3,733)_ = 7.197, *p* < 0.001 and Expt. 2: *F*_(3,715)_ = 5.27, *p* = 0.004]. No effect was found for treatment [Expt.1 males: *F*_(1,429) =_ 0.0804, *p* = 0.778, females: *F*_(1,303)_ = 0.156, *p* = 0.696; Expt.2: males *F*_(1,399)_ = 1.063, *p* = 0.316, females *F*_(1,356)_ = 0.143, *p* = 0.709].

### Binge Ethanol during Adolescence Increases Locomotor Activity without Altering Basal Anxiety

**FIGURE 1:**
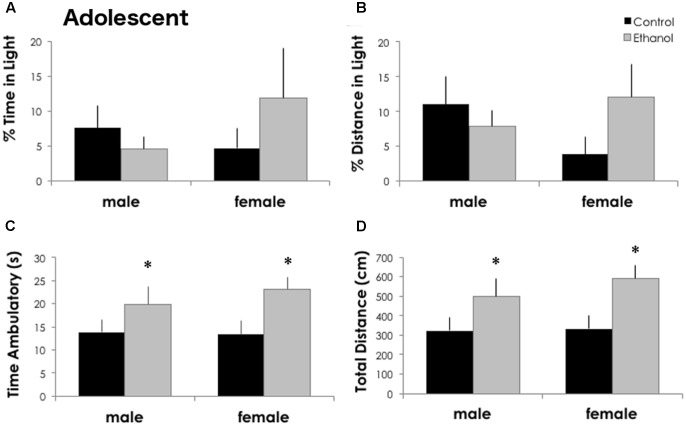
Binge ethanol in adolescence increases locomotor activity in adolescence. Ethanol treatment did not alter the percent time in the light **(A)** or the percent distance traveled in the light **(B)** in the light–dark box 24 h after the last ethanol dose at PND 43 (*n* = 7–10/group). Time spent ambulatory **(C)** and total locomotor distance traveled **(D)** in the 5-min test were significantly increased in ethanol exposed males and females. Data is presented as mean +/– SEM. ^∗^*p* < 0.05, main effect of treatment by Two-Way ANOVA.

Twenty-four hours after the last dose of ethanol, anxiety-like behavior in the light–dark assay was not altered by ethanol exposure in DBA/2J adolescents. The percent time in the light [*F*_(1,32)_ = 0.273, *p* = 0.606] and the percent distance traveled in the light [*F*_(1,32)_ = 0.516, *p* = 0.478] were not significantly different between treatment groups (**Figure 1**) or sexes [% time: *F*_(1,32)_ = 0.287, *p* = 0.596 and % distance: *F*_(1,32)_ = 0.178, *p* = 0.677]. Locomotor activity, however, was significantly increased in ethanol exposed adolescents. The total distance traveled and the time spent in ambulation was significantly increased in ethanol exposed mice [main effect of treatment: *F*_(1,32)_ = 7.688, *p* = 0.010 and *F*_(1,32)_ = 6.043, *p* = 0.020]. Both males and females exposed to ethanol showed a robust, significant increase in locomotor activity (54 and 77% increase in total distance, respectively) and ambulatory time (43 and 72% increase) although there were no differences between males and females.

### Adolescent Ethanol Exposure Reduces Sensitivity to High Doses of Ethanol

**FIGURE 2:**
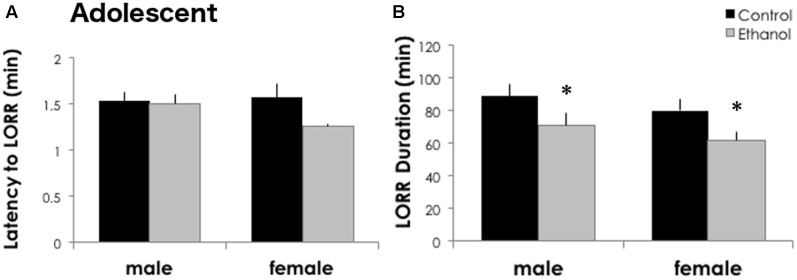
Adolescent ethanol reduces ethanol sensitivity in adolescent DBA/2J mice. **(A)** DBA/2J males (*n* = 9/group) and females (*n* = 6–7/group) take a similar amount of time to lose their righting reflex after high doses of ethanol. **(B)** Both binge treated males and females showed a shorter LORR duration after a high dose of ethanol at PND 46. Data is presented as mean +/– SEM. ^∗^*p* < 0.05, main effect of treatment by Two-Way ANOVA.

Intermittent binge ethanol during adolescence reduced the sedative/hypnotic response to ethanol in DBA/2J males and females at PND 46. The latency to lose the righting reflex was similar between treatment groups [*F*_(1,30)_ = 2.726, *p* = 0.110] and sexes [**Figure 2A**, *F*_(1,30)_ = 0.972, *p* = 0.333]. However, treatment with 4 g/kg ethanol gavage from postnatal day 29 to 42 reduced the time sedated in the loss of righting reflex test (**Figure 2B**). We found a significant main effect of treatment in DBA/2J mice for LORR duration [*F*_(1,30)_ = 6.032, *p* = 0.021], but no significant effect of sex [*F*_(1,30)_ = 1.419, *p* = 0.244] or interaction between sex and ethanol treatment [*F*_(1,30)_ = 0.00006, *p* = 0.994]. DBA/2J adolescents had a 20 and 22.7% reduction in sleep time following a history of ethanol exposure in males and females, respectively.

### Binge Ethanol during Adolescence Impairs Memory in Adulthood

**FIGURE 3:**
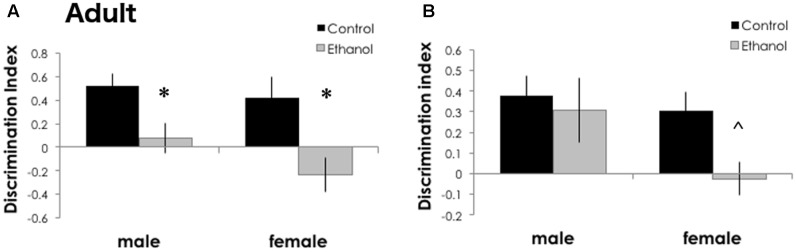
Recognition memory was impaired in adult mice after binge ethanol in adolescence. After a 5-min delay between training and testing **(A)**, ethanol-exposed DBA/2J adult mice (*n* = 6–11/group) had a zero or negative discrimination index, indicating a failure to recognize a novel object, or failure to remember a familiar object. After a 1-h delay **(B)**, DBA/2J adult female mice had a trend to a lower discrimination index. Data is presented as mean +/– SEM. ^∗^*p* < 0.05, main effect of treatment by Two-Way ANOVA. ˆ*p* = 0.07, a trend for a significant effect of treatment.

To estimate the long-term effects of binge ethanol during a period of frontal cortical development, we measured “PFC-mediated” short term memory and “perirhinal/hippocampal-mediated” longer term memory using the novel object recognition test (Warburton and Brown, 2015). In DBA/2J adults exposed to binge ethanol during adolescence, recognition memory was decreased in ethanol exposed males and females after a 5-min delay [**Figure 3A**, *F*_(1,29)_ = 15.403, *p* < 0.001]. Only a trend toward a decrease in recognition memory was found to be affected by ethanol exposure after a 1-h delay, primarily driven by females [**Figure 3B**, *F*_(1,25)_ = 3.477, *p* = 0.074]. We did not find significant effects of sex at either inter-interval delay [5-min delay: *F*_(1,29)_ = 0.321, *p* = 0.156 and 1-h delay: *F*_(1,25) =_ 0.180, *p* = 0.207]. Nor did we find a significant interaction between treatment and sex [5-min delay: *F*_(1,29)_ = 0.088, *p* = 0.453 and 1-h delay: *F*_(1,25)_ = 0.693, *p* = 0.413]. Together, this data suggests that adolescent binge ethanol in DBA/2J mice may selectively damage the frontal cortical connections, because novel object recognition was decreased after a short (5 min) but not a long (1 h) delay. We may also be uncovering a sex-specific effect, where females are more strongly impacted by adolescent ethanol than males. There were no significant differences between groups for time investigating the objects during the training sessions (*p* > 0.05, data not shown).

### Ethanol-Induced Anxiolysis Is Unaffected by Adolescent Ethanol in Adult Mice

**FIGURE 4:**
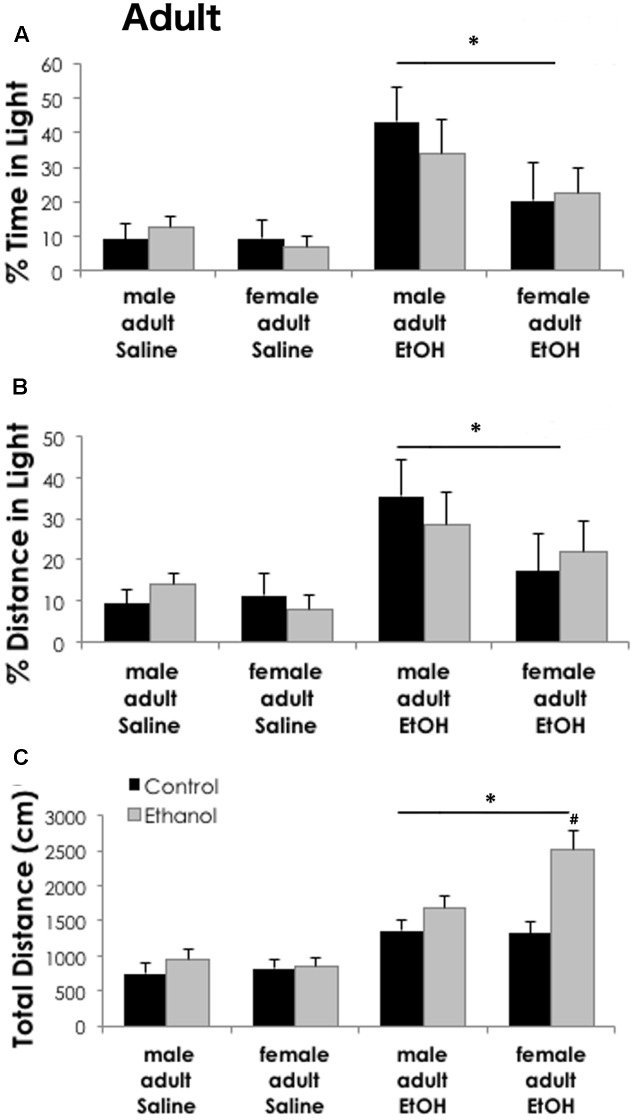
Binge ethanol does not alter ethanol-induced anxiolysis in adulthood. Ethanol (2 g/kg i.p.) significantly increased time spent in the light **(A)** and distance traveled in the light **(B)** as compared to adult saline treated mice (*n* = 5–10/group). Sex differences or prior exposure to ethanol did not modify anxiety-like behavior in the light–dark box. **(C)** Total distance traveled during the task was higher in ethanol treated mice. Females previously exposed to ethanol during adolescence had significantly higher locomotor activity as compared to all other groups. Data is presented as mean +/– SEM. ^∗^*p* < 0.05, main effect of ethanol injection by Three-Way ANOVA. ^#^*p* < 0.05 for interaction between adolescent exposure, ethanol injection and sex by Three-Way ANOVA.

Anxiety-like behaviors in the light–dark box following saline or ethanol (2.0 g/kg i.p.) treatment were similar in adult mice exposed to binge ethanol as adolescents versus controls. Percent time and distance in the light were not significantly different between sexes [time: *F*_(1,64)_ = 2.99, *p* = 0.089; distance: *F*_(1,64)_ = 2.077, *p* = 0.115] or adolescent treatment groups [time: *F*_(1,64)_ = 0.079, *p* = 0.780; distance: *F*_(1,64)_ = 0.002, *p* = 0.962]. Acute ethanol (2 g/kg i.p.) significantly increased both the percent time [*F*_(1,64)_ = 12.45, *p* < 0.001] and distance traveled [*F*_(1,64)_ = 9.32, *p* = 0.003] in the light compartment as compared to saline treatment, indicating a significant ethanol anxiolytic-like response (**Figure 4**). Prior ethanol exposure during adolescence did not interact with these measures. Total distance traveled during the assay, however, was significantly higher in mice treated with acute ethanol [main effect of LD box treatment, *F*_(1,64)_ = 48.57, *p* < 0.001] as compared to saline treated counterparts. Some attributes of juvenile locomotor activation may persist into adulthood. When tested as adults in the LD box, mice exposed to adolescent binge ethanol showed increased locomotor activity when injected with acute 2 g/kg ethanol, as compared to the saline treated mice [**Figure 4C**, main effect of adolescent exposure, *F*_(1,64)_ = 11.96, *p* = 0.001]. A significant interaction among all three factors [adolescent exposure, LD box treatment and sex; *F*_(1,64)_ = 4.23, *p* = 0.044] revealed that in ethanol treated adults, females exposed to binge ethanol had greater locomotion than control females, while males did not.

### Adolescent Binge Ethanol Increases Ethanol Sensitivity in Adulthood

**FIGURE 5:**
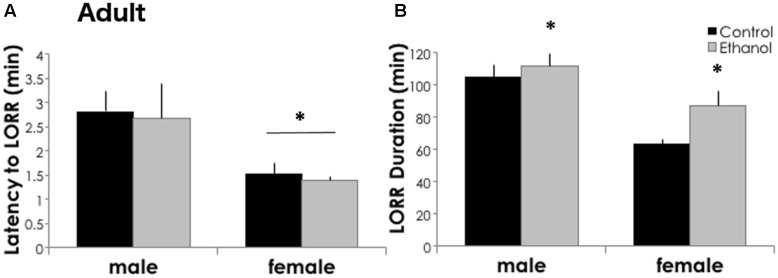
Ethanol sensitivity is greater in ethanol-exposed mice in adulthood. **(A)** Latency to lose the righting reflex was significantly shorter in adult females (*n* = 13/group) as compared to males (*n* = 19–20/group). ^∗^*p* < 0.05, main effect of sex by Two-Way ANOVA. But did not differ between treatment groups. **(B)** In adulthood, adolescent ethanol exposure increased LORR duration as adults. Data is presented as mean +/– SEM. ^∗^*p* < 0.05, main effect of treatment by Two-Way ANOVA.

Three weeks after the last ethanol binge, mice exposed to ethanol as adolescents were more sensitive to the sedative/hypnotic effects of high dose ethanol as compared to controls. The latency to lose the righting reflex was similar between treatment groups [*F*_(1,61)_ = 0.013, *p* = 0.910], but shorter in females [**Figure 5A**, *F*_(1,61)_ = 6.604, *p* = 0.013]. In adults, pretreatment with adolescent ethanol increased the time sedated in the loss of righting reflex test. Adults had a 6.5 and 36.9% increase in sleep time following a history of ethanol exposure in males and females, respectively (**Figure 5B**). We found a significant main effect of treatment for LORR duration [*F*_(1,61)_ = 4.427, *p* = 0.040], and a significant effect of sex [*F*_(1,61)_ = 19.633, *p* < 0.001], but no significant interaction between the two [*F*_(1,61)_ = 1.095, *p* = 0.300]. Overall, females had a shorter LORR duration than males.

### Genomic Expression Analysis in PFC Reveals Changes in Myelination and Histone Methylation in Adolescents

In experiment 2, behaviorally naïve tissue from the PFC was collected 24 h (at PND 43) and 3 weeks (at PND 66) after the last ethanol binge. Total RNA was analyzed for gene-level expression differences using Mouse Transcriptome Arrays v1.0. Two complementary analyses were conducted to interrogate differential gene expression at each age. Twenty-four hours after the last ethanol binge, using a two-way ANOVA in limma with treatment and sex as factors, 493 transcript IDs were significantly altered as a main effect of treatment at *p* < 0.01 (Supplementary Table 1). 393 transcript IDs were significantly altered by sex and 244 transcript IDs were differentially expressed in the interaction between treatment and sex (Supplementary Table 1). Gene Ontology over-representation analysis identified six categories involved in oligodendrocyte development and myelination as the primary Biological Processes altered by adolescent binge ethanol (Supplementary Table 2). For transcript IDs significant for the interaction between sex and adolescent treatment, Gene Ontology analysis only identified two over-represented cellular components: ER chaperone component and smooth ER. When comparing gene expression between adolescent males vs. females, most of the differentially expressed genes either resided on the Y chromosome (*Ddx3y*, *Eif2s3y*, *Kdm5d*, *Uty*), or are known to escape X-inactivation (*Ddx3x*, *Eif2s3x*, *Kdm5*c, *Kdm6a*) in mice (Yang et al., 2010). Over-represented Gene Ontology categories (Supplementary Table 2) reflect their processes, such as histone demethylase activity, angiotensin catabolic processes in blood, cell adhesion and regulation of gap junction assembly.

In adult mice, 626 transcript IDs were altered by binge ethanol in adolescence at *p* < 0.01 (Supplementary Table 3). 256 transcript IDs were significantly altered as a main effect of sex and 242 transcript IDs were significantly for the interaction between treatment and sex (Supplementary Table 3). Gene Ontology analysis only identified two over-represented molecular functions as significantly over-represented for a main effect of treatment: Beta-catenin binding and transcription factor activity (Supplementary Table 4). Gene Ontology comparisons for a main effect of sex in adult mice mainly revealed changes in oxidoreductase activity and histone demethylase activity due to the lysine demethylases that reside on the Y-chromosome, or are subject to escape from X-inactivation (Supplementary Table 4). The interaction between adolescent ethanol treatment and sex in adult mice revealed many significantly over-represented GO categories that were quite disparate including lipid binding, inflammatory response, regulation of ERK cascades, regulation of vesicle mediated transport and learning and memory.

We performed a second analysis using the *S*-score probe-level algorithm which we have previously shown to have increased sensitivity for differential expression analysis (Zhang et al., 2002; Kennedy et al., 2006). For this analysis, data was collapsed over sex since to increase the power to detect differences between ethanol treatment versus controls and to focus on lasting differences following binge ethanol. In adolescents, 24 h after the last ethanol dose, 1812 transcript IDs were significantly altered by ethanol in the PFC (Supplementary Table 5). Importantly, 300 transcript IDs out of the 493 transcript IDs significantly altered by binge ethanol in the TAC/limma analysis were also included in the *S*-score results. These included all of the myelin-related genes described above. Gene Ontology analysis of the *S*-score results also identified a very cohesive set of significantly over-representation molecular functions and biological processes related to histone demethylase activity, specifically at H3K9 and H3K36 residues (Supplementary Table 6). Ingenuity Pathway analysis identified two novel networks that contained many of these histone demethylases (**Supplementary Figure S1**).

In adults, 3 weeks after the last binge ethanol, 1553 transcript IDs were significantly altered by ethanol (Supplementary Table 5) using the *S*-score algorithm. Only a few GO categories were significantly over-represented (Supplementary Table 6). These were mainly involved in glutamate receptor signaling, specifically at the AMPA receptor, regulation of RAS signal transduction and synaptic signaling. The top novel network from Ingenuity Pathway Analysis (**Supplementary Figure S2**) contained seven of the eight AMPA-related genes downregulated in adult brains after adolescent binge ethanol.

To assess genes that were persistently regulated long-term following adolescent binge ethanol, we intersected the *S*-score analysis gene list significantly altered by ethanol in adolescents with the list obtained from adults. Surprisingly, only 49 transcript IDs were in common between the two age groups (Supplementary Table 5). These genes were primarily involved in RNA polymerase II activating transcription factor binding, regulation of cytosolic calcium, and negative regulation of apoptosis were the major themes. Ingenuity Pathway Analysis identified canonical pathways similar to the GO analysis including G-protein coupled receptor signaling and cAMP mediated signaling (Supplementary Table 6).

### Adolescent Binge Ethanol Reduces Myelin-Related Gene Expression

**FIGURE 6:**
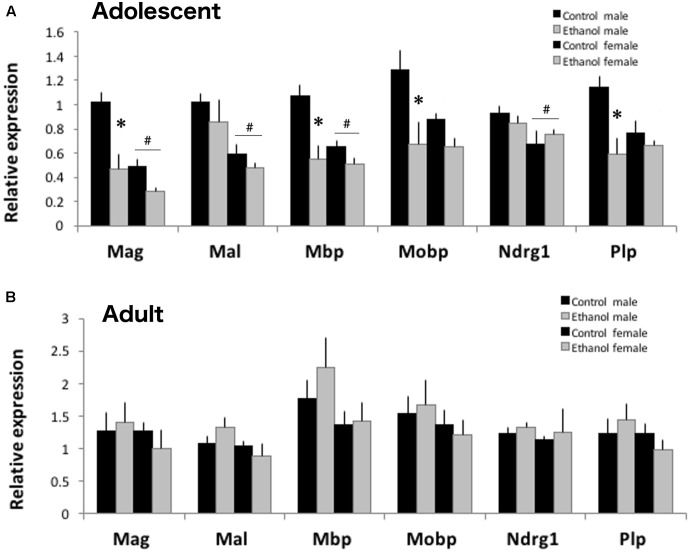
Binge ethanol during adolescents alters mRNA expression of myelin-related genes. **(A)** Binge ethanol decreased many myelin-related genes including *Mag*, *Mbp*, *Mobp* and *Plp* mRNA in male and female DBA/2J PFC by qPCR (*n* = 4–6, ^∗^*p* < 0.05, interaction between treatment and sex by Two-Way ANOVA). In adolescence, DBA/2J males have more myelin-related mRNA than females (^#^*p* < 0.05, main effect of sex by Two-Way ANOVA). **(B)** In adulthood, prior binge ethanol did not alter myelin-related gene expression. Sex differences were not found in adults. Data is presented as mean +/– SEM.

Both the *S*-score analysis and the TAC two-way ANOVA analysis contained 300 transcript IDs in common for treatment responses in adolescent animals. Among those, four myelin-related genes, *Mag*, *Mbp*, *Mobp*, and *Plp* were significantly reduced in both analyses. We confirmed reduction of these myelin-related gene expression in adolescent males and females after binge ethanol using qPCR (**Figure 6A**). Sex differences in myelin-related gene expression were noted in adolescent PFC. Females had lower expression of *Mag* [*F*_(1,19)_ = 19.478, *p* < 0.001], *Mal* [*F*_(1,19)_ = 15.635, *p* < 0.001], *Mbp* [*F*_(1,19)_ = 8.966, *p* = 0.009], and *Ndrg1* [*F*_(1,19)_ = 5.789, *p* = 0.029] than males. Binge ethanol exposure during adolescence significantly reduced expression of *Mag* [*F*_(1,19)_ = 19.478, *p* < 0.001], *Mbp* [*F*_(1,19)_ = 17.805, *p* < 0.001], *Mobp* [*F*_(1,19)_ = 10.531, *p* = 0.005], and *Plp* [*F*_(1,19)_ = 11.941, *p* = 0.003]. Effects appear to be stronger in males, as a significant interaction between treatment and sex was found for *Mag* [*F*_(1,29)_ = 4.464, *p* = 0.05], *Mbp* [*F*_(1,29)_ = 6.147, *p* = 0.025], and *Plp* [*F*_(1,19)_ = 5.581, *p* = 0.031], where expression was lower in ethanol exposed males versus control males. Expression of two myelin related genes not significantly altered by ethanol in our microarray analysis, *Mal* and *Ndrg1*, were also unchanged by binge ethanol using qPCR [*Mal*: *F*_(1,29)_ = 1.812, *p* = 0.197 and *Ndrg1*: *F*_(1,29)_ = 0.0017, *p* = 0.968]. To see if these gene expression changes persisted, we also surveyed myelin expression by qPCR in adult PFC at PND66. None of the myelin-related genes were significantly altered by adolescent ethanol exposure [*Mag*: *F*_(1,17)_ = 0.064, *p* = 0.804; *Mal*: *F*_(1,17)_ = 0.111, *p* = 0.743; *Mbp*: *F*_(1,17)_ = 0.584, *p* = 0.458; *Mobp*: *F*_(1,17)_ = 0.0014, *p* = 0.971; *Ndrg1*: *F*_(1,17)_ = 0.383, *p* = 0.546; and *Plp*: *F*_(1,17)_ = 0.018, *p* = 0.896] or sex [*Mag*: *F*_(1,17)_ = 0.573, *p* = 0.462; *Mal*: *F*_(1,17)_ = 0.294, *p* = 0.108; *Mbp*: *F*_(1,17)_ = 2.992, *p* = 0.106; *Mobp*: *F*_(1,17)_ = 1.027, *p* = 0.328; *Ndrg1*: *F*_(1,17)_ = 0.201, *p* = 0.661; and *Plp*: *F*_(1,17)_ = 1.167, *p* = 0.298; **Figure 6B**] in adult animals, consistent with the microarray results.

### Histone Methylation of H3K36 Is Reduced by Binge Ethanol

**FIGURE 7:**
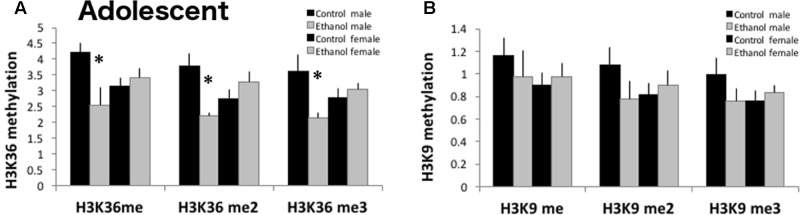
H3K36 methylation is decreased in ethanol exposed adolescent males. **(A)** Using ELISAs to measure global methylation in PFC (*n* = 3–4/group), binge ethanol decreased H3K36me, H3K36me2 and H3K36me3 in DBA males (^∗^*p* < 0.05, interaction between treatment and sex by Two-Way ANOVA). **(B)** H3K9 methylation (mono- di- or tri-) was not significantly altered by binge ethanol (*n* = 3–4/group). Data is presented as mean +/– SEM.

Since genes involved in histone demethylase activity, specifically at H3K9 and H3K36, were reduced in ethanol exposed adolescent mice, we quantified global histone methylation protein levels for mono-, di-, and tri-methylation at H3K9 and H3K36 (**Figure 7**) in a separate cohort of mice. At PND 43, 24 h after the last gavage treatment, all three H3K36 methylation protein levels were significantly decreased in ethanol treated males as compared to control males [significant interaction: H3K36me *F*_(1,13)_ = 7.006, *p* < 0.024; H3K36me2 *F*_(1,13)_ = 10.904, *p* = 0.008; H3K36me3 *F*_(1,13)_ = 7.438, *p* = 0.021]. Global levels of H3K9 mono-, di-, or tri-methylation were not altered by adolescent binge ethanol [H3K9me *F*_(1,15)_ = 0.160, *p* = 0.697; H3K9me2 *F*_(1,15)_ = 0.105, *p* = 0.752; H3K9me3 *F*_(1,15)_ = 0.581, *p* = 0.461].

## Discussion

Early adolescent experience with alcohol durably changes brain structure, alters the behavioral properties of ethanol, and can increase risk for developing alcohol use disorder. These studies were conducted to begin to identify the molecular changes occurring after binge ethanol within the developing PFC and to determine the molecular changes persisting in the adult brain. This work is the first genome-wide analysis of PFC gene expression responses to adolescent binge ethanol. We report that binge ethanol during the adolescent period decreases myelin-related gene expression and expression of genes involved in histone methylation in the PFC. In adulthood, glutamate signaling is decreased in the PFC of mice exposed to binge ethanol during adolescence. Interestingly, some transcripts involved in G-protein signaling altered after the last binge ethanol session were persistently changed in adulthood. These gene expression profiles were associated with differences in ethanol behavioral responses.

In mice exposed to repeated adolescent ethanol by gavage, we noted immediate and long-lasting changes in ethanol behavioral sensitivity (**Figures 2**, **5**). Similar to prior reports (Hefner and Holmes, 2007; Linsenbardt et al., 2009; Broadwater et al., 2011), adolescent DBA/2J males and females were less sensitive to the sedative/hypnotic effects of ethanol following binge ethanol in the loss of righting reflex task. Adults previously exposed to binge ethanol were more sensitive and took longer to recover than controls. As compared to adults, adolescent rats and mice have shorter sedation after a high dose of ethanol and are less sensitive to the sedative/hypnotic effects (Hefner and Holmes, 2007; Linsenbardt et al., 2009; Broadwater et al., 2011). These developmental differences were attributed to differences in ethanol pharmacokinetics in ethanol-naïve DBA/2J mice (Linsenbardt et al., 2009). Although, differences in adolescent versus adult ethanol metabolism were not always found (Hefner and Holmes, 2007). We did not measure ethanol pharmacokinetics in our model and thus we cannot exclude the possibility that either age-related differences in ethanol metabolism or potential tolerance from repeated binge exposures are contributing to these differences in ethanol sedation. We also did not compare ethanol metabolism differences between males and females. Others, however, have reported no sex differences in ethanol metabolism in DBA (Linsenbardt et al., 2009) or C57BL/6 mice (Gorin-Meyer et al., 2007; Linsenbardt et al., 2009). Although, we cannot say whether sex differences in ethanol metabolism can account for the behavioral differences discussed below, we feel that it is unlikely that they play a major role here. We did not directly compare adolescents and adults since the assays were conducted about a month apart. However, we found that prior exposure to binge ethanol made adolescent and adult responses to ethanol more extreme than controls. This could suggest that ethanol exposure primes or sensitizes the system and strengthens the age-appropriate behavioral responses. We do not believe that the reduced sensitivity observed in adolescents is simply due to tolerance since the loss of righting test was performed 4 days after the last ethanol binge.

Additionally, in our model, adolescent binge ethanol may promote prolonged locomotor sensitization. While testing for anxiety 24 h after the last binge, ethanol exposed mice traveled farther in the light–dark apparatus than controls. In adulthood, a priming injection of 2 g/kg ethanol while testing for ethanol-induced anxiolysis, increased locomotor activity in females with a history of binge ethanol. In rodents, adolescents are more sensitive to low dose ethanol’s locomotor stimulating and locomotor sensitization effects than adults (Lopez et al., 2003; Hefner and Holmes, 2007; Stevenson et al., 2008). Swiss mice exposed to ethanol as adolescents, especially at high doses, have enhanced locomotor stimulant effects as compared to controls and these effects persisted for 3 weeks after the last ethanol exposure (Quoilin et al., 2012, 2014). Our behavioral studies in adult animals were similarly initiated 24 days after the last adolescent gavage ethanol treatments. Ethanol-induced locomotor sensitization is associated with ethanol’s reinforcing effects and related to its addictive properties (Wise and Bozarth, 1987). While we did not directly measure the effects of ethanol-induced locomotor activity in adolescents, we propose that binge ethanol exposure has sensitized the mice and as adolescents, the mice may be displaying increased locomotor activity during the light–dark test. This early life exposure has also possibly produced a sex-specific long-term ethanol sensitization such that in adulthood, a low-dose ethanol challenge causes greater locomotor responses in female animals (**Figure 4C**). Future studies will be needed to directly test this effect and the persistence of locomotor sensitization.

At the dose used in these studies, DBA/2J mice did not display withdrawal-induced anxiety. Twenty-four hours after the last ethanol binge, basal anxiety in the light–dark apparatus was similar between ethanol-exposed and control mice. Others have reported withdrawal-induced anxiety in adolescent rats (Pandey et al., 2015; Kyzar et al., 2016), although this is not consistently found (Kiefer et al., 2003; Lee et al., 2016; Morais-Silva et al., 2016). Indeed, some have suggested that adolescent mice are resilient to early ethanol withdrawal (Lee et al., 2016) and it is possible that binge ethanol in adolescence only produces lasting withdrawal-induced anxiety in rats and not mice. Importantly, as discussed above, we observed increased locomotor activity in ethanol-exposed mice. Locomotor differences can confound the interpretation of anxiety-phenotypes in the light–dark box. Additionally, control mice spent only about 8–10% of their time in the light, suggesting a floor effect for anxiety-phenotypes in the light–dark box. Thus, our lack of withdrawal-induced anxiety must be carefully interpreted.

To test the hypothesis that adolescent binge ethanol disrupts PFC signaling, attention or memory, we used the novel object recognition task to assess recognition memory. This task is based on innate novelty seeking and thus requires no external motivation or reward and little training (Antunes and Biala, 2012). This task is sensitive to disruptions in PFC and perirhinal cortex signaling (Seamans et al., 1995) and shows memory deficits following adolescent ethanol (Montesinos et al., 2014; Beaudet et al., 2016). Adult DBA/2J mice exposed to binge ethanol as adolescents had deficits in recognition memory that were stronger with the short, PFC-mediated inter-trial delay (**Figure 3**). Females showed a trend for deficits with the longer, perirhinal-mediated delay. Together with the locomotor activity, this could suggest that females may be more sensitive than males to the prolonged effects of adolescent ethanol exposure. Alternatively, as the novel object recognition task is based on novelty-seeking, mice that spend less time investigating a novel object may be experiencing neophobia. Future studies investigating the effects of binge ethanol on neophobia and/or other anxiety-related tasks are needed to investigate this possibility. Importantly, using different ethanol exposure paradigms and different cognition tasks, others have reported that adolescent rats and mice exposed to ethanol display deficits in reversal learning and in object recognition that persist into adulthood (Coleman et al., 2011, 2014; Vargas et al., 2014; Beaudet et al., 2016) and may be associated with changes in cortical volume (Coleman et al., 2011, 2014) and myelin ultrastructure (Vargas et al., 2014). Variations in myelin are also associated with subtle differences in cognitive and behavioral performance (Perrin et al., 2008; Paus, 2010). Additionally, deficits in novel object recognition have been linked to *Shank3* loss of function and associated reduced glutamate neurotransmission and long-term potentiation (Yang et al., 2012). Our genomic analyses suggest that DBA/2J mice, 3 weeks after the last ethanol dose, also had decreased *Shank3* expression, and may have reduced glutamate and AMPA signaling in the frontal cortex. Little has been reported on the role of glutamate signaling within the PFC of adolescents. One recent study has suggested that glutamate signaling may underlie adolescent vulnerability to binge drinking (Agoglia et al., 2015). Another study reported increased dendritic spine density without alterations in glutamatergic protein expression the prelimbic cortex in adulthood following adolescent binge ethanol (Trantham-Davidson et al., 2017). However, in adult rodents and alcoholics, glutamate signaling and expression of the metabotropic glutamate receptor 2 (mGluR2) was markedly downregulated in the PFC of post-dependent rats (Meinhardt et al., 2013), as well as in post-mortem tissue from mPFC of human alcoholics (Heilig et al., 2017). Together with the reduced myelin expression, decreases in glutamatergic neurotransmission from the frontal cortex to other brain regions could be one mechanism through which ethanol reduced recognition memory.

Ethanol has repeatedly been linked to alterations in PFC myelin gene expression (Lewohl et al., 2000; Liu et al., 2004; Kerns et al., 2005; Wolstenholme et al., 2011b; Farris and Miles, 2013). Growing interest in the possible role of myelin in mediating long-term consequences of adolescent ethanol exposure has been fueled by human neuroimaging findings in white matter changes in the PFC (De Bellis et al., 2005; Medina et al., 2008; Luciana et al., 2013). Intermittent ethanol during adolescence in rats reduced corpus callosum size and increased degraded myelin (Vargas et al., 2014). We also observed reductions in myelin-related gene expression in the frontal cortex of adolescent mice exposed to binge ethanol (**Figure 6**). However, differences in myelin-related gene expression did not persist into adulthood, 3 weeks after the last ethanol dose. It is plausible that myelin expression may recover over time after cessation of ethanol exposure since continued abstinence from alcohol partially reverses the white matter loss in uncomplicated alcoholics (Shear et al., 1994; Pfefferbaum et al., 1995). Ongoing studies are assessing whether myelin protein expression or structure could be altered persistently into adulthood with our adolescent binge model.

Our bioinformatics analysis of genomic data also identified genes involved in histone demethylase activity, specifically genes that regulate H3K9 and H3K36 methylation, to be significantly downregulated in the PFC of adolescents after binge ethanol. Indeed, 7 out of 10 genes in the GO:0010452 pathway: histone H3K36 methylation and 5 out of 8 genes in the GO:0032454: histone demethylase (H3K9 specific) pathway were significantly decreased in the adolescent PFC. Interpreting the role of these epigenetic modifications is complicated by the fact that lysine can be mono-, di-, or tri-methylated, and the degree of methylation can differentially influence gene expression. For example, H3K9me2 is enriched in transcriptionally silent euchromatic domains, while H3K9me3 mediates heterochromatin formation by forming a binding site for HP1 and participates in gene silencing at euchromatic sites (Lehnertz et al., 2003). Each epigenetic enzyme identified in our analysis specifically catalyzes modification of a particular amino acid on a particular histone and exhibits specificity toward the degree of methylation at that site (**Supplementary Figure S1**). H3K9me3 was recently identified as the histone mark underlying transcriptional changes in glutamate or GABA synapses on oligodendrocyte precursor cells (OPCs) as they mature into myelin-forming oligodendrocytes (Liu et al., 2015). In OPCs, H3K9me3 is increased in genes from neuronal lineages, in genes related to the regulation of membrane excitability, or in repressive protein complexes. Decreased expression of this mark in binge ethanol adolescents as well as reduced glutamate signaling could indicate that oligodendrocytes are not maturing into myelin-forming cells and this may be one mechanism through which ethanol is eliciting its effects to repress oligodendrocyte differentiation and thus retard frontal cortex development in binge drinking adolescents.

H3K36 methylation, alternatively, is associated with active transcription. H3K36me is enriched in coding regions of active genes, while H3K36me2 has a role in double strand break repair (Jha and Strahl, 2014). H3K36me3 is displaced by RNA pol II, and acts as a mark for HDAC binding to prevent runaway transcription (Butler and Dent, 2012). Reduction of these marks, as found in the PFC of adolescents after binge ethanol, could release the brakes on RNA polymerase II and increase aberrant, cryptic transcription at alternative promoter sites and transcriptional dysregulation.

Recent studies in alcohol and other drug dependence are beginning to identify the role of histone methylation and other epigenetic changes in addiction-related phenotypes. In adolescent rats, intermittent ethanol upregulated histone acetyl transferase (HAT) activity and histone acetylation in the PFC (Pascual et al., 2012). Systemic administration of HDAC inhibitors further increase PFC HAT activity, but only in adolescents. Adults were not affected by HDAC inhibition suggesting that these epigenetic modifications have temporal resolution that is important developmentally. In the developing amygdala, HDAC activity and HDAC2 levels leading to deficits in histone (H3K9) acetylation in the central nucleus of the amygdala (CeA) and persisted in to adulthood (Pandey et al., 2015).

A growing consensus is developing to suggest a role for histone methylation in addiction. For example, adolescent intermittent ethanol increases H3K4me2 in the promoter regions of cFos, Cdk5, and FosB in the PFC (Pascual et al., 2012). H3K9me1 and Prdm2, the enzyme responsible for mono-methylation of H3K9, were decreased in the PFC of dependent rats (Barbier et al., 2016). PFC viral vector mediated reduction of Prdm2 in non-dependent rats increased ethanol drinking even in the presence of quinine adulteration displaying phenotypes characteristics of alcohol dependence. Additionally, H3K9me2 is decreased in the nucleus accumbens after repeated cocaine or morphine (Sun et al., 2012), but H3K9me3 is upregulated after acute cocaine (Maze et al., 2010) and decreases upon withdrawal. The histone methyl-transferase, G9a, may be involved in cocaine-induced plasticity and morphine-associated behaviors (Maze et al., 2010; Sun et al., 2012), further suggesting that histone methylation may also be relevant for addiction. Further studies are necessary to determine the specific loci where these modifications are acting in our model and their role in the long-term expression of ethanol sensitivity and cognitive decline.

Epigenetic marks and the enzymes that add or remove these modifications fluctuate in expression throughout development. We hypothesize that ethanol-induced perturbation of histone methylation during crucial developmental periods, such as adolescence, may cause widespread genetic dysregulation as seen with myelin and glutamate signaling-related gene expression in our studies here. Such responses could affect normal developmental trajectories and cause the persistence and/or emergence of ethanol behavioral pathology in adulthood. Future studies will seek to causally connect discrete epigenetic modifications with specific gene expression and behavioral sequelae resulting from adolescent ethanol exposure.

## Author Contributions

The study was conceived, designed, executed analyzed, and written by JW. qPCR studies were conducted by TM. GH and SA adapted the *S*-score algorithm to work for the MTA 1.0 microarrays. MM provided resources, experimental interpretation and critical review of the manuscript.

## Conflict of Interest Statement

The authors declare that the research was conducted in the absence of any commercial or financial relationships that could be construed as a potential conflict of interest.

## Footnotes

1 www.ingenuity.com

## Supplementary Material

FIGURE S1 Novel gene networks altered by binge ethanol in adolescent mice. Two novel gene networks generated by Ingenuity Pathway Analysis identified genes involved in histone methylation. The table reflects genes in the Gene Ontology analysis, GO: 0032454 histone demethylase activity (H3-K9 specific) or GO:0010452 histone H3-K36 methylation that were significantly altered by binge ethanol using the *S*-score analysis.

FIGURE S2 Novel gene network altered by binge ethanol and persistently altered in adulthood. A representative novel gene network generated by Ingenuity Pathway Analysis of genes persistently altered by adolescent binge ethanol. This network is enriched in genes involved in AMPA receptor signaling.
