# Supplementary material for: Intermittent Ethanol during Adolescence Leads to Lasting Behavioral Changes in Adulthood and Alters Gene Expression and Histone Methylation in the PFC
Source: Front Mol Neurosci. 2017 Sep 26;10:307. doi: 10.3389/fnmol.2017.00307 (PMC5622951; doi:10.3389/fnmol.2017.00307)
Supplement: FIGURE S1 — Novel gene networks altered by binge ethanol in adolescent mice. Two novel gene networks generated by Ingenuity Pathway Analysis identified genes involved in histone methylation. The table reflects genes in the Gene Ontology analysis, GO: 0032454 histone demethylase activity (H3-K9 specific) or GO:0010452 histone H3-K36 methylation that were significantly altered by binge ethanol using the S-score analysis. [file Image_1.PDF]

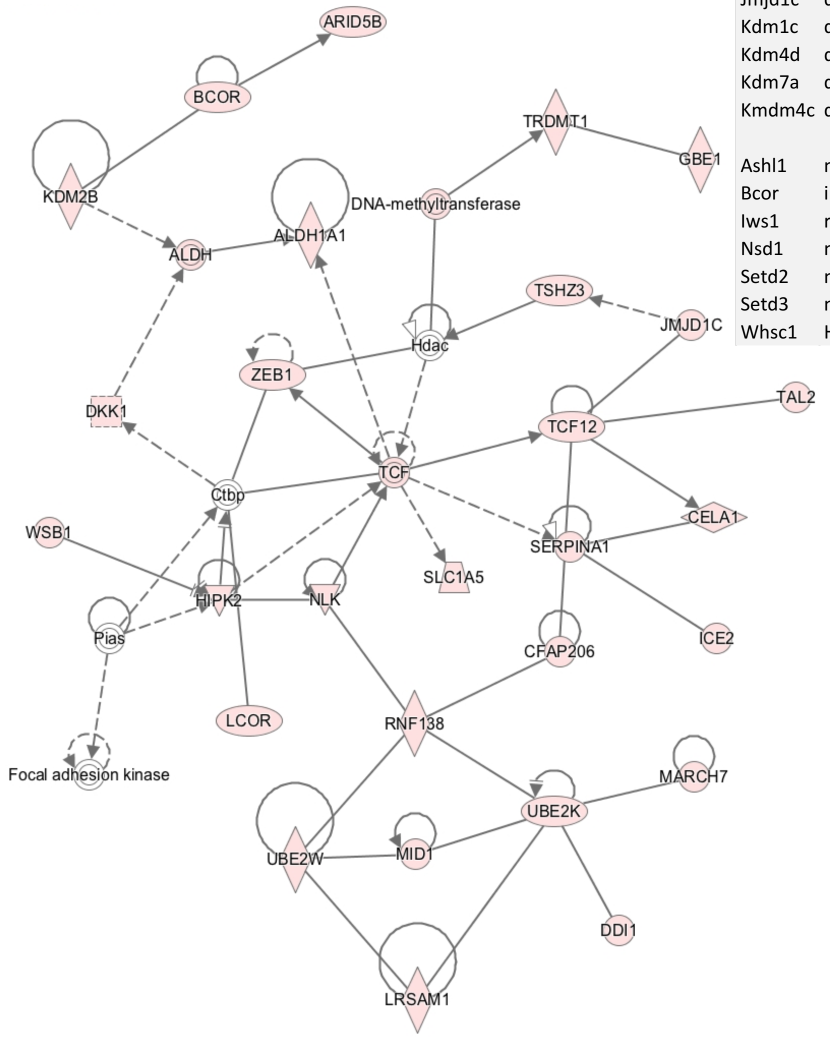

© 2000-2016 QIAGEN. All rights reserved.

Gene Symbol	Action	S-score q-value	limma Etoh main effect p-value
Jmjd1c	demethylase at H3K9	0.026	0.200
Kdm1c	demethylase at H3K4 and H3K9	0.030	0.029
Kdm4d	demethylates H3K9me2 and me3	0.019	0.060
Kdm7a	demethylates H3K9me2 and H3K27me2	0.042	0.034
Kmdm4c	demethylates H3K9me3 and H3K36me3	0.022	0.067
Ashl1	methyltransferase at H3K36me and me2	0.041	0.198
Bcor	inhibits H3K4me3 and H3K36me2	0.048	0.016
Iws1	recruits Setd2 for mRNA splicing	0.019	0.014
Nsd1	methylase for H3K36 and H4K20	0.019	0.026
Setd2	methylates H3K36me3	0.017	0.017
Setd3	methylates H3K4me and H3K36me	0.041	0.089
Whsc1	H3K27 methyltransferase	0.012	0.038

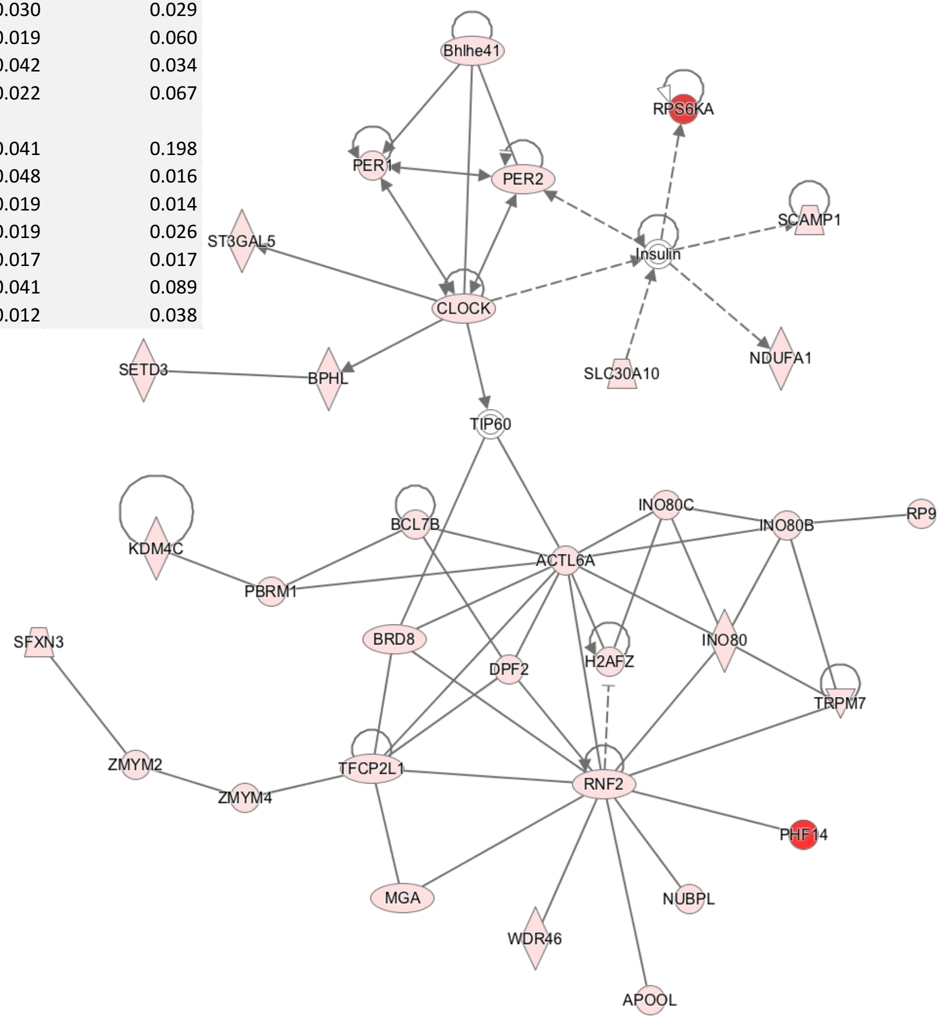

© 2000-2016 QIAGEN. All rights reserved.
