# Supplementary figures and images for: Intermittent Ethanol during Adolescence Leads to Lasting Behavioral Changes in Adulthood and Alters Gene Expression and Histone Methylation in the PFC
Source: Front Mol Neurosci. 2017 Sep 26;10:307. doi: 10.3389/fnmol.2017.00307 (PMC5622951; doi:10.3389/fnmol.2017.00307)

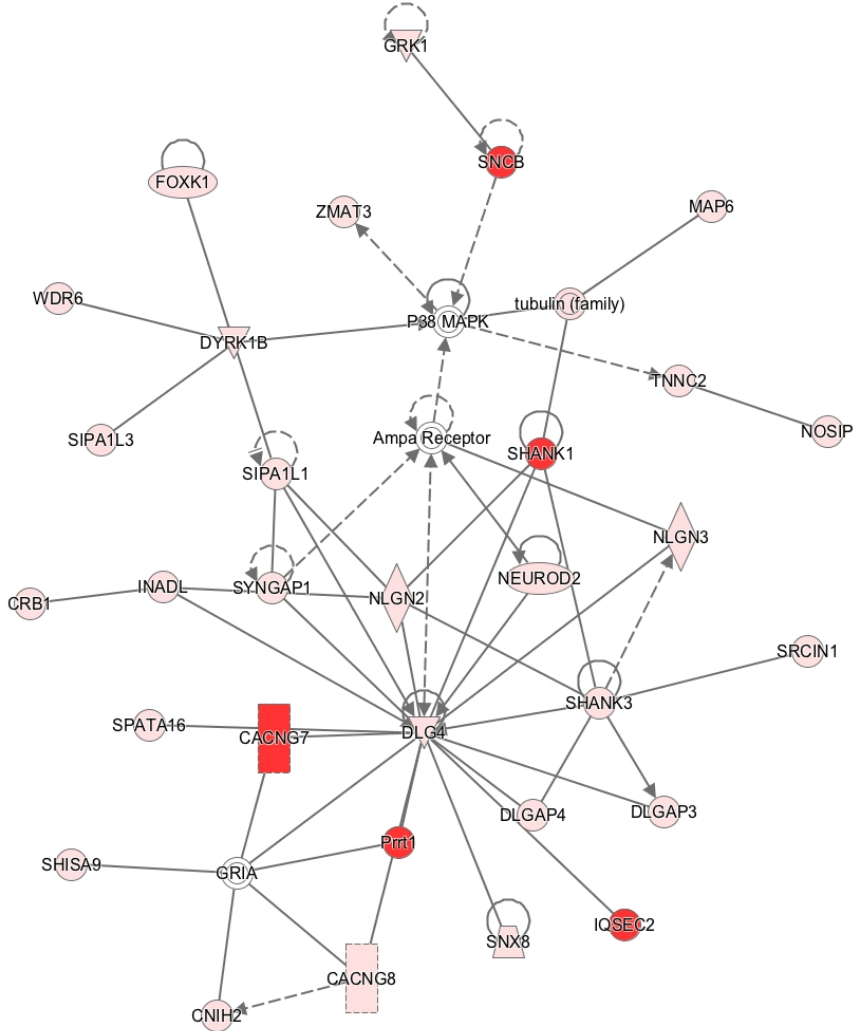

Supplement: FIGURE S2 — Novel gene network altered by binge ethanol and persistently altered in adulthood. A representative novel gene network generated by Ingenuity Pathway Analysis of genes persistently altered by adolescent binge ethanol. This network is enriched in genes involved in AMPA receptor signaling. [file Image_2.PDF]
